# Competitive DNA transfection formulation via electroporation for human adipose stem cells and mesenchymal stem cells

**DOI:** 10.1186/1480-9222-14-7

**Published:** 2012-04-18

**Authors:** Michael Flanagan, Jeffrey M Gimble, Gang Yu, Xueqing Xia, Bruce A Bunnell, Shulin Li

**Affiliations:** 1 Department of Comparative Biomedical Sciences, Louisiana State University, Skip Bertman Drive, Baton Rouge, LA 70803, USA; 2 Pennington Biomedical Research Center, 6400 Perkins Rd., Baton Rouge, LA 70808, USA; 3 Department of Pediatrics Research, The University Texas MD Anderson Cancer Center, Graduate School of Biomedical Sciences, 1515 Holcombe, Houston, TX, USA; 4 Director, Center for Stem Cell Research and Regenerative Medicine, Tulane University School of Medicine, 1430 Tulane Ave, New Orleans, LA, USA; 5 Department of Pediatrics Research, The University of Texas MD Anderson Cancer Center, The University of Texas Graduate School of Biomedical Sciences at Houston, Houston, TX 77030, USA

**Keywords:** Electroporation, Formulation, Stem cells, Transfection, Cell therapy

## Abstract

**Background:**

Adipose stem cells have a strong potential for use in cell-based therapy, but the current nucleofection technique, which relies on unknown buffers, prevents their use.

**Results:**

We developed an optimal nucleofection formulation for human adipose stem cells by using a three-step method that we had developed previously. This method was designed to determine the optimal formulation for nucleofection that was capable of meeting or surpassing the established commercial buffer (Amaxa), in particular for murine adipose stem cells. By using this same buffer, we determined that the same formulation yields optimal transfection efficiency in human mesenchymal stem cells.

**Conclusions:**

Our findings suggest that transfection efficiency in human stem cells can be boosted with proper formulation.

## Background

Cell-based therapies have great potential for the treatment of genetic disorders as well as currently incurable diseases. Stem cells, the most attractive candidate for such therapy, have been tested in the treatment of leukemias [1,2] and in the regrowth of damaged tissue [3]. Adipose-derived stem cells (ASCs) have recently been isolated [4] and characterized [5]. ASCs are a relatively abundant and easily isolated pluripotent cell line, which makes them a promising candidate as a vehicle for stem cell therapy [4,6,7]. ASCs can be modified to differentiate into various cell lineages, including adipogenic, chondrogenic, and osteogenic cell lines [8], as well as into myoblasts and endothelial cells [5]. ASCs have also demonstrated the ability to home to certain types of tumors [9], which makes them a viable option for antitumor cell therapy.

In a previous study, we developed a method for optimizing formulations to aid in the delivery of plasmid DNA in the process of nucleofection [10]. Although nucleofection is an effective form of nonviral transfection for many types of stem cells [11], its therapeutic use is limited by the availability of secret formulations developed by the commercial vendor Amaxa, which must be purchased directly from the vendor. Our devised method offers a three-step plan for determining an optimal transfection formulation generated from known chemicals. The use of this formulation is more economical and in many cases surpasses the formulation developed by Amaxa. Notably, our method resulted in the use of pluronic-block copolymers for the development of an optimal nucleofection formulation for murine ASCs.

In this study, we applied the method we developed in our previous study [10] and explored the optimal nucleofection formulation for human ASCs (hASCs) and human mesenchymal stem cells (hMSCs). This study looks even further into members of the pluronic-block copolymer family and their effect on transfection efficiency in hASCs.

## Results and discussion

### Initial determination of optimal buffer, electroporation program, and polymer

**Figure 1:**
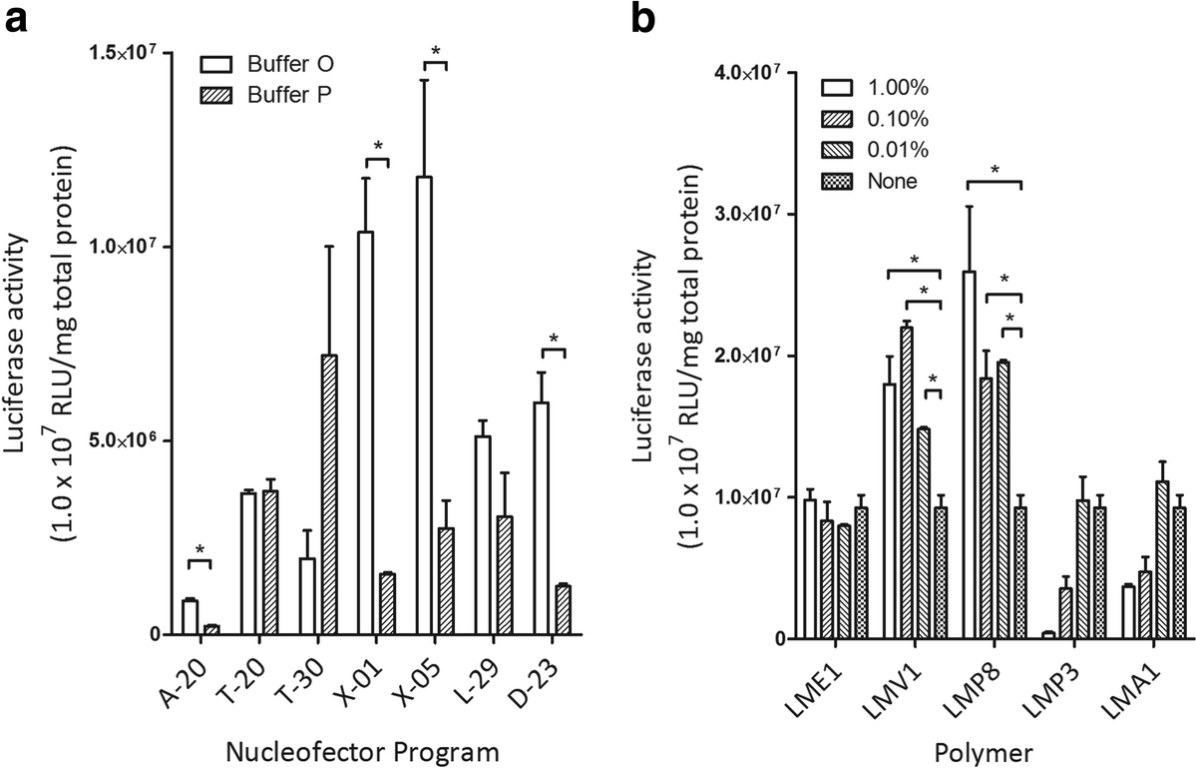
**First two steps in the selection of electroporation formulation for human adipose-derived stem cells (ASCs)**. Error bars expressed as mean × SEM (n = 3). *, indicating a significant difference was detected at p < 0.05. (**a**) Step 1 of buffer optimization. 1 × 10E6 cells were used for each cell transfection in 100 μL indicated buffers and different programs with use of Amaxa nucleofector. (**b**) Step 2 of buffer optimization. The key for polymer abbreviations is in the Methods section. Differentiation of each polymer was tested. None, optimum buffer without addition of any polymer.

To determine the optimal nucleofection formulation for increasing transfection efficiency of hASCs by using an Amaxa nucleofection device, we decided to use known cell transfection electroporation buffers as a starting point. Following our optimized nucleofection method developed previously [10], we initially chose two buffers: OptiMEM and pulsing buffer. To determine the nucleofection program that would yield the highest effectiveness, we used the following seven programs, as outlined in the Amaxa Nucleofector Optimization Protocol: A-20, T-20, T-30, X-01, X-05, L-29, and D-23. The results of this first step are displayed in Figure 1a. Although it appears the optimal program is X-05, program X-01 plus OptiMEM buffer yields a comparable and more consistent increase in transfection efficiency (Figure 1a), which was selected for further transfection analysis.

After determining the optimal buffer and electroporation program, we determined whether the addition of any polymers would further increase the effectiveness of transfection. We tested five different polymers, specifically LME1, LMV1, LMP8, LMP3, and LMA1, as outlined in the Methods section. Our results of step two, displayed in Figure 1b, showed that both LMV1 and LMP8 produced the strongest increase in transfection efficiency and were significantly better than other polymers (p < 0.05). LMP8 was selected for futher analysis because it had a higher rate of transfection than LMV1 (though not significant) and is consistent with our findings with murine ASCs [10].

### Exploration of pluronic-block copolymers

**Figure 2:**
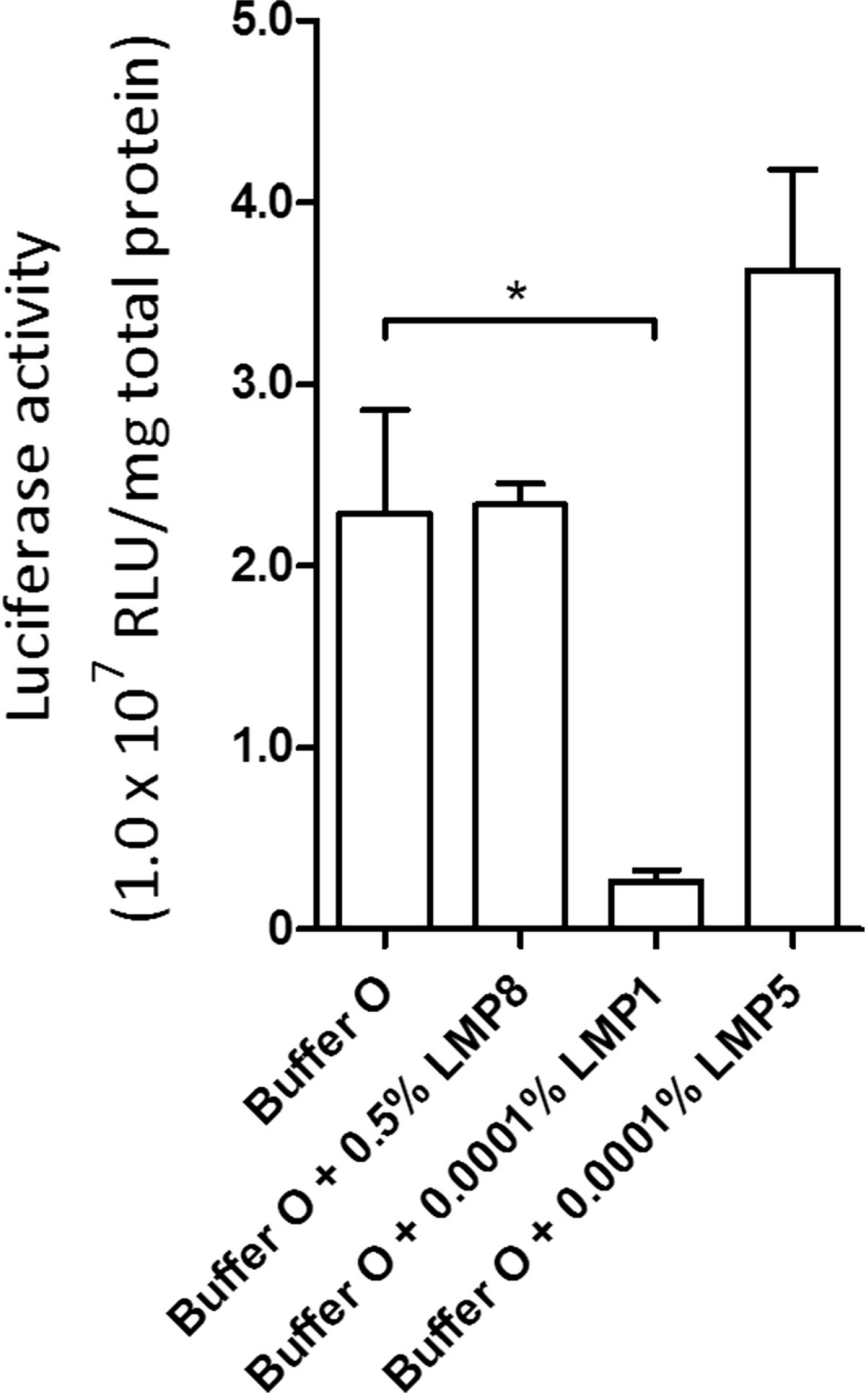
**Comparison of increase in transfection from polymers from pluronic family for human adipose-derived stem cells (ASCs)**. Error bars expressed as mean × SEM (n = 3). *, indicating a significant difference was detected at p < 0.05. The key for polymer abbreviations can be found in the Methods section. Cells were nucleofected with program X-01, as determined in the first step.

We previously optimized the transfection buffer for murine ASCs and found that pluronic-block copolymers caused the strongest increase in transfection. There are numerous types of pluronic-block copolymers that vary in molecular weight and hydrophilic properties. LMP1, which is hydrophobic, bears a smaller hydrophilic region, has a much lower molecular weight, and produced a much stronger increase in transfection than LMP8 did in murine ASCs. Different from murine ASCs, LMP1 failed to increase the effectiveness of transfection in hASCs. However, we observed a marked increase in transfection efficiency with the use of LMP5, which has a low molecular weight that is similar to that of LMP1, is much more hydrophilic, and has a hydrophile-to-hydrophobe ratio that is similar to that of LMP8 (Figure 2).

**Figure 3:**
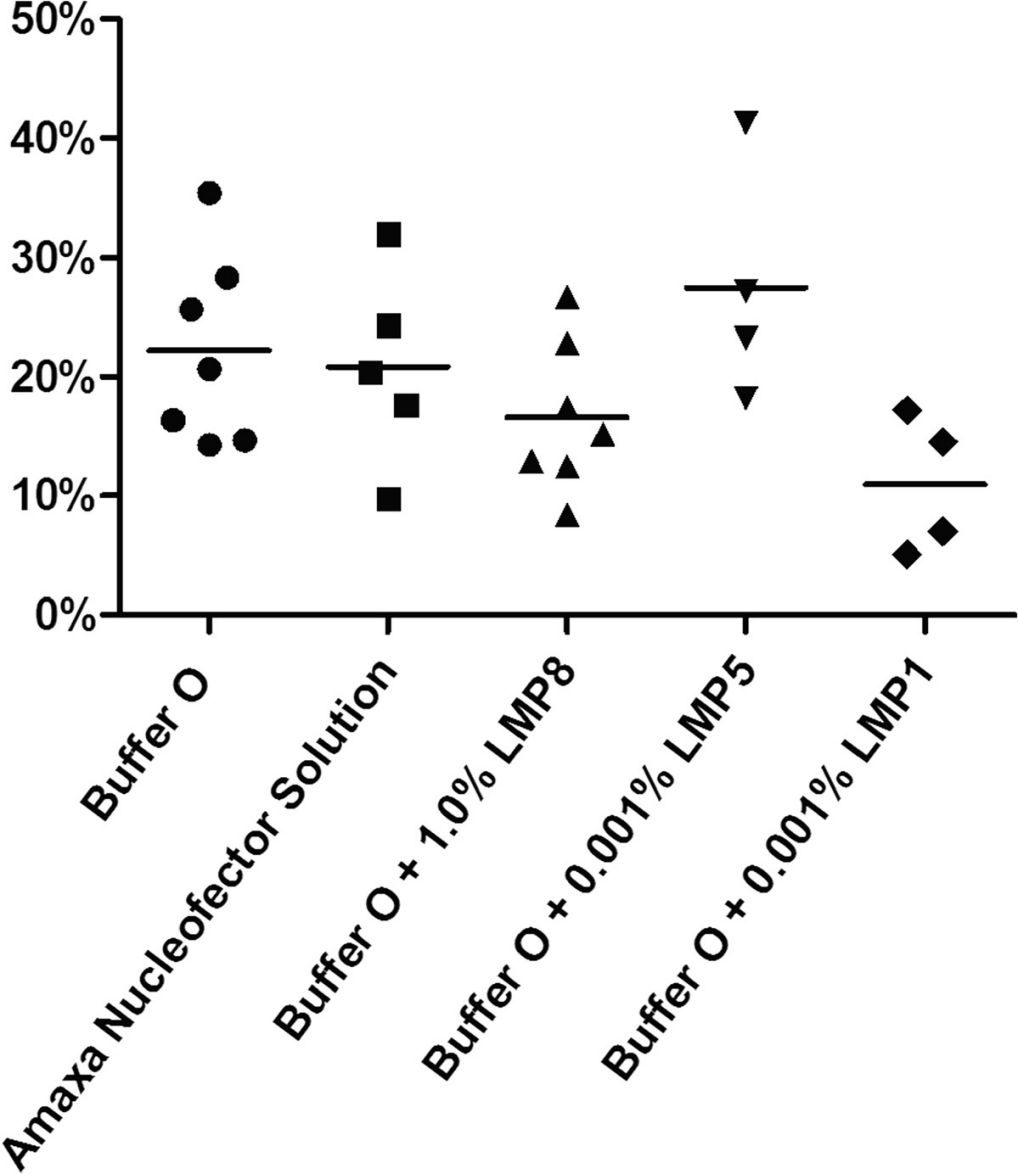
**Comparison of percentage of adipose-derived stem cells (ASCs) positive for green fluorescent protein, as measured by FACS**. Cells treated with Amaxa nucleofector solution were nucleofected with program U-23. All other cells were nucleofected with program X-01, as determined in the first step. Bar indicates mean transfection efficiency.

To increase the fidelity of our discovery, each polymer was repeatedly tested. Unlike other members of the pluronic-block copolymer family, which consistently fail to increase transfection efficiency, LMP5 efficiency persistently trended higher than that of Buffer O alone (Figure 3).

### Observation of ASCs under scanning electron microscopy

**Figure 4:**
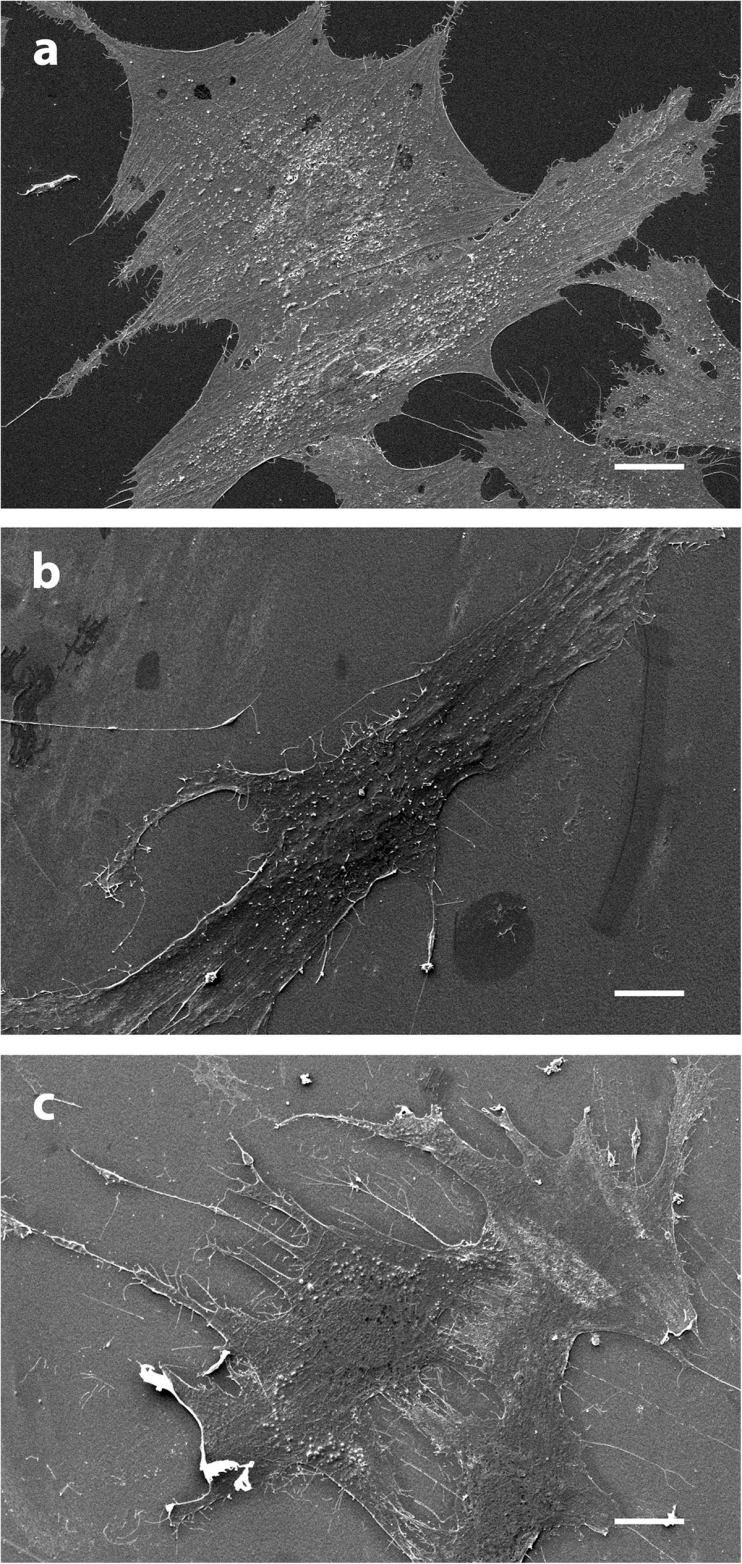
**Scanning electron micrograph of human adipose-derived stem cells (hASCs)**. (**a**) hASCs in DMEM cell culture medium. (**b**) hASCs in buffer O. (**c**) hASCs in buffer P.

DNA or cell formulations are generally considered to change the electric, chemical, and biological properties of cells, which can increase cell transfection efficiency. We wanted to analyze possible underlying mechanisms by which a formulation increases cell transfection efficiency. We used scanning electron microscopy (SEM) to observe morphological changes in the ASCs in the presence of our cell formulations. We incubated the seeded ASCs in DMEM, OptiMEM, and Pulsing Buffer. The SEM images in Figure 4a lack the projected small white dots near the nuclei (mitochondria), which were present when cells were incubated in cell culture medium DMEM. These projected mitochondria were absent in cells incubated in OptiMem (buffer O) and Pulsing (buffer P) (Figure 4). These results demonstrate the association between the effective buffer for transfection and mitochondria projection.

### Effects of pluronic-block copolymers on hMSCs

**Figure 5:**
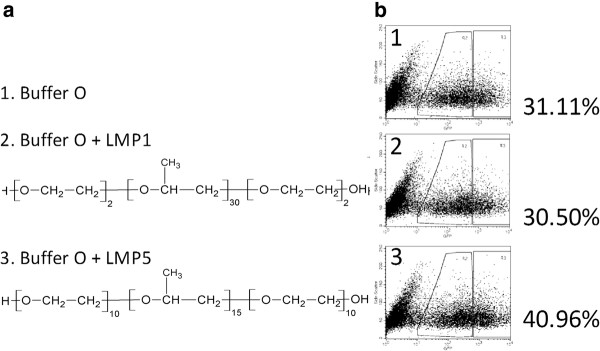
**Efficiency of cellular transfection of human mesenchymal stem cells (MSCs) between Buffer O and two pluronic-block copolymers**. (**a**) Chemical composition of modifiers used for in part **b**. (**b**) Detection of green fluorescent protein (GFP)-positive cells using FACS, with percentage of hASCs positive for expression of GFP.

Of the pluronic-block copolymers, LMP1 showed the greatest increase in transfection efficiency for ASCs of murine origin [10]. We tested the effectiveness of pluronic-block copolymers on hMSCs, which are phenotypically similar to hASCs [12]. We compared various pluronic-block copolymers that were previously optimized for both hASCs and murine ASCs, along with Buffer O (Figure 5a), which was shown to be the best buffer for both cell lines. We found that LMP5 yielded the same increase in transfection efficiency-40% from the base efficiency of 30%-in hMSCs as in hASCs (Figure 5b).

### Comparison of transfection efficiencies

Our goal was to increase the rate of transfection of hASCs and hMSC. We sought to increase efficiency through modification of the formulation. With use of pluronic-block copolymers in combination with optimal buffers, we were able to successfully increase transfection efficiency up to 40%, which successfully outperformed the Amaxa buffer (32%) in our experiments (Figure 3). This number is much lower than the 73% [11] achieved in hMSCs when Amaxa buffers were used by others. However, the 73% may have been overstated [13].

Another study [14], which used the Amaxa MSC nucleofector solution, claimed a 40%-60% transfection rate with hASCs. However, it should be noted that, in the previously mentioned study, the transfection rate was determined through a count of fluorescent cells versus cells present in bright field imaging [14]. Our data showed that analysis by flow cytometry provides a more accurate representation of the true rate of transfection. Zaragosi provides the best comparison, with 24-hour transfection rates at 54.7% [15].

The main purpose of this study was to develop a simple and publicly known cell formulation that would replace the secret Amaxa buffer for efficient transfection of hASCs and hMSCs. We sought to accomplish this through the use of known buffers in combination with polymers. As observed in previous studies [10], the pluronic-block copolymer family shows the most consistent promise of boosting transfection efficiency. Pluronic-block copolymers, which are composed of an internal polyoxypropylene (hydrophobic) chain bordered by external polyoxyethylene (hydrophilic) chains, have been previously shown to have some use in gene therapy [16]. The most notable observation was evidence that different species of the pluronic-block copolymer family provide the optimal boost in nucleofector efficiency specific to each cell line. The variation of species within this group is determined by two factors: the ratio of hydrophobic to hydrophilic chains and the total molecular weight of the species [16].

In our previous study, we determined LMP1 to have a greater effect on transfection efficiency than LMP8 for murine ASCs. This study illustrates that LMP5 is more effective than LMP1 or LMP8 for two independent types of human stem cells. The LMP1 species has both a lower molecular weight and a lower hydrophile-to-hydrophobe ratio than does LMP8. The LMP5 species has a hydrophile-to-hydrophobe ratio more similar to LMP8 and a similar molecular weight to LMP1 (~2 KDa). The effectiveness of LMP5, but not of LMP8 or LMP1, suggests that both molecular weight and polarization are critical for effectively transfecting human cells via nucleofection.

### Potential mechanism of transfection

The mechanism of electroporation is not well understood. The current thought is that electroporation creates small pores in the plasma membrane, allowing large molecules to enter the cytosol through simple diffusion [17,18]. Originally, polymers in solution were believed to bind to DNA and assist in transport across the membrane [19]. However, we showed in earlier studies that the addition of pluronic-block copolymers offered no difference in transfection effectiveness when first combined with cells or with DNA [10]. It is worth noting that the increase in transfection results from the influence of the pluronic molecules on the cell itself. In our previous study, we looked at morphologic changes to the overall structure of the cell in different buffer conditions. Using SEM, we found that the nucleus of the cell projected more toward the plasma membrane, which offers a possibility for the transport of macromolecules directly into the nucleus [10]. For hASCs, we did not observe the same nuclear projection that was noted with murine ASCs. Instead, we noted an increase in mitochondrial activity around the nuclear region, suggesting that the mitochondria play a role in boosting DNA transfection. This should be further explored in future studies.

## Conclusion

hASCs and hMSCs have a similar phenotype [5,12], so polymers that boost transfection efficiency of one cell type should produce a similar boost in the other. Our initial comparison of pluronic-block copolymers in hMSCs shows that the same concentration of LMP5 produces the same increase in transfection efficiency in both cell types. This suggests that the same mechanism is at work to boost transfection efficiency for human stem cell lines. Further study is needed to understand this relationship and to determine whether any other cell types that are boosted in this fashion.

## Methods

### Cell culture

The hASCs were isolated from human adipose tissue by collagenase digestion and culture expansion in DMEM/F12 Ham's Medium, 10% FBS, 1% antibiotic/antimycotic according to published methods [4]. Isolation of adipose-derived adult stem cells from human liposuction aspirates and surgically excised adipose depots was approved by Pennington Biomedical Reearch Center, Louisiana State University system with IRB number 23040.

### Buffers and polymers

OptiMEM (Buffer O) is a commercial buffer that was purchased from Invitrogen (Carlsbad, CA) and stored at 4°C. Pulsing buffer (Buffer P), consisting of 125 mM KCl, 15 mM NaCl, 3 mM glucose, 25 mM HEPES, and 1.2 mM MgCl_2 _(pH7.4), is made from a 10× homemade stock solution, filtered, and stored at 4°C. After preparation, the solution is filtered through a 0.2-μm filter and stored at room temperature.

LMA1 stock was made to a 10% (wt/vol) of Poly-L-glutamic acid (mw 15-50 kDa, Sigma-Aldrich, St Louis, MO) in MQ-H_2_O. LME1 (polyethylene glycol, mw 8 kDa, Amresco, Solon, OH), LMP8 (poloxamer 188, mw 8 kDa, Spectrum Chemicals, New Brunswick, NJ), LMP1 (poloxamer 181, mw 2 kDa, Spectrum Chemicals), LMP5 (poloxamer 95, mw 1.8 kDa, Sigma-Aldrich), and LMV1 (polyvinylpyrrolidone, mw 40 kDa, Fisher Scientific, Pittsburg, PA) stocks were all made to 20% (wt/vol) in MQ-H_2_O. LMP3 (Pop313, donated from Expression Genetics, Huntsville, AL) was made to a stock concentration of 0.01% (wt/vol) in MQ-H_2_O. All stock buffers were stored at 4°C and were kept for 1 month.

### Electroporation of plasmid DNA

Cells were pelleted and resuspended in 100 μL transfection buffer at a concentration of 1.0 × 10^6 ^cells/100 μL and then transferred to a sterile 3-mm Amaxa nucleofection cuvette. Cells were incubated with 2 μg reporter gene encoding plasmid DNA. The reporter genes, GFP (green florescence protein) and luciferase, were driven by CMV promoter at the 5'-end and terminated by the human growth hormone polyadenylation signal at the 3' end. The size of GFP and luciferase plasmid DNA are 4.7 and 4.3 kb, respectly. Cells were electroporated with use of the appropriate nucleofection program. Nucleofected cells were then rinsed with 500 μL of sterile culture medium and transferred to the well of a sterile 12-well plate. Cells were incubated at 37°C for 24 hours before analysis.

### Flow cytometry

For flow cytometry analysis, GFP plasmid DNA was transfected into the targeted cells using the indicated formulation. GFP positive cells (% of total cells) were detected using flow cytometry. Before flow cytometry was performed, cells were harvested by trypsinization and resuspended in 1000 μL of PBS. Some of the cell suspension (500 μL) was removed and fixed in PBS + 1.0% formaldehyde solution before analysis. Cells were measured by a BD FACSCalibur (BD Biosciences; San Diego, CA).

### Luciferase and protein assays

After incubation, cells were lysed and assayed for luciferase activity (Cell lysis kit, Promega; Madison, WI; part# E4030). Briefly, cell pellets were rinsed with PBS and lysed with 200 μL of lysis reagent with use of the freeze-thaw method. Cell lysates were kept at 4°C during analysis and stored at -20°C when not in use. Next, 20 μL of cell lysates (after spining) were transferred to a 96-well plate. Luciferase activity was measured by using a Packard LumiCount (Perkin Elmer; Boston, MA). All luciferase activity was normalized to the protein with use of the following equation: [(luciferase activity in RLU) × 200]/(per *μ*g protein).

Protein assays were conducted by using a commercially purchased BCA protein assay kit (Thermo Scientific; Rockford, IL; Product # 23227). Briefly, 4 μL of cell lysate prepared from luciferase assay was transferred to a clear 96-well plate. Protein levels were measured by a Packard SpectraCount (Perkin Elmer). Protein assay measurements were calibrated to a BSA standard with a maximum of 40 μg and analyzed by I-Smart 2.0 (Perkin Elmer).

### Electron microscopy

5 × 10^4 ^hASCs were seeded onto 13-mm round Thermanox cover slips (Thermo Fisher Scientific, Rochester, NY) placed in a sterile 24-well plate. Cells were grown until they reach 40%-50% confluence. All growth medium was removed from the wells, and cells were incubated at room temperature for 10 minutes in one of the following solutions before processing for SEM: DMEM, Buffer O, Buffer P, and 1/2 saline nucleofector solution. Briefly, cells were fixed in 1.25% glutaraldehyde and 2% formaldehyde in 0.1 M sodium cacodylate (CAC) buffer for 1 hour at room temperature. Cells were then washed in 0.1 M CAC buffer, treated with 1% osmium tetroxide in 0.1 M CAC, and washed again. Cells were then dehydrated in ethanol before platinum-coating.

## Abbreviations

ASCs: Adipose-derived stem cells; hASCs: Human ASCs; hMSCs: Human mesenchymal stem cells; MSCs: Mesenchymal stem cells; SEM: Scanning electron microscopy.

## Competing interests

The authors declare that they have no competing interests.

## Authors' contributions

MF, primary one who have conducted the cell transfection and analysis; JMG and GY, primary ones who provided all hASC; XX, primary one who provided plasmid DNA; SL, primary one who developed the ideas and experimental design. All authors read and approved the final manuscript.

## References

[B1] Lee ST Jang JH Cheong JW Kim JS Maemg HY Hahn JS Ko YW Min YH Treatment of high-risk acute myelogenous leukaemia by myeloablative chemoradiotherapy followed by co-infusion of T cell-depleted haematopoietic stem cells and culture-expanded marrow mesenchymal stem cells from a related donor with one fully mismatched human leucocyte antigen haplotype Br J Haematol 2002 118 1128 1131 10.1046/j.1365-2141.2002.03767.x 12199796

[B2] de Lima Prata K Delgado Orellana M Cunha De Santis G Kashima S Fontes AM de CÃ¡ssia Viu Carrara R Vianna Bonini Palma P Neder L Tadeu Covas D Effects of high-dose chemotherapy on bone marrow multipotent mesenchymal stromal cells isolated from lymphoma patients Exp hematol 2010 38 4 292 300 10.1016/j.exphem.2010.01.006 20138957

[B3] Dupont KM Sharma K Stevens HY Boerckel JD Garcia AJ Guldberg RE Human stem cell delivery for treatment of large segmental bone defects Proc Natl Acad Sci USA 2010 107 3305 3310 10.1073/pnas.0905444107 20133731 PMC2840521

[B4] Katz AJ Llull R Hedrick MH Futrell JW Emerging approaches to the tissue engineering of fat Clin Plast Surg 1999 26 587 603 viii 10553215

[B5] Gimble J Guilak F Adipose-derived adult stem cells: isolation, characterization, and differentiation potential Cytotherapy 2003 5 362 369 10.1080/14653240310003026 14578098

[B6] Morizono K De Ugarte DA Zhu M Zuk P Elbarbary A Ashjian P Benhaim P Chen IS Hedrick MH Multilineage cells from adipose tissue as gene delivery vehicles Hum Gene Ther 2003 14 59 66 10.1089/10430340360464714 12573059

[B7] Gimble JM Katz AJ Bunnell BA Adipose-derived stem cells for regenerative medicine Circ Res 2007 100 1249 1260 10.1161/01.RES.0000265074.83288.09 17495232 PMC5679280

[B8] Bunnell BA Flaat M Gagliardi C Patel B Ripoll C Adipose-derived stem cells: isolation, expansion and differentiation Methods 2008 45 115 120 10.1016/j.ymeth.2008.03.006 18593609 PMC3668445

[B9] Josiah DT Zhu D Dreher F Olson J McFadden G Caldas H Adipose-derived stem cells as therapeutic delivery vehicles of an oncolytic virus for glioblastoma Mol Ther 2010 18 377 385 10.1038/mt.2009.265 19904233 PMC2839314

[B10] Flanagan M Gimble JM Yu G Wu X Xia X Hu J Yao S Li S Competitive electroporation formulation for cell therapy Cancer Gene Ther 2011 18 579 586 10.1038/cgt.2011.27 21660061 PMC3238913

[B11] Aluigi M Fogli M Curti A Isidori A Gruppioni E Chiodoni C Colombo MP Versura P D'Errico-Grigioni A Ferri E Baccarani M Lemoli RM Nucleofection is an efficient nonviral transfection technique for human bone marrow-derived mesenchymal stem cells Stem Cells 2006 24 454 461 10.1634/stemcells.2005-0198 16099993

[B12] Gronthos S Franklin DM Leddy HA Robey PG Storms RW Gimble JM Surface protein characterization of human adipose tissue-derived stromal cells J Cell Physiol 2001 189 54 63 10.1002/jcp.1138 11573204

[B13] Wang YH Ho ML Chang JK Chu HC Lai SC Wang GJ Microporation is a valuable transfection method for gene expression in human adipose tissue-derived stem cells Mol Ther 2009 17 302 308 10.1038/mt.2008.267 19066595 PMC2835049

[B14] Wolbank S Peterbauer A Wassermann E Hennerbichler S Voglauer R van Griensven M Duba HC Gabriel C Redl H Labelling of human adipose-derived stem cells for non-invasive in vivo cell tracking Cell Tissue Bank 2007 8 163 177 10.1007/s10561-006-9027-7 17063258

[B15] Zaragosi LE Billon N Ailhaud G Dani C Nucleofection is a valuable transfection method for transient and stable transgene expression in adipose tissue-derived stem cells Stem Cells 2007 25 790 797 17158239 10.1634/stemcells.2006-0235

[B16] Kabanov AV Lemieux P Vinogradov S Alakhov V Pluronic block copolymers: novel functional molecules for gene therapy Adv Drug Deliv Rev 2002 54 223 233 10.1016/S0169-409X(02)00018-2 11897147

[B17] Mir LM Nucleic acids electrotransfer-based gene therapy (electrogenetherapy): past, current, and future Mol Biotechnol 2009 43 167 176 10.1007/s12033-009-9192-6 19562526

[B18] Zaharoff DA Henshaw JW Mossop B Yuan F Mechanistic analysis of electroporation-induced cellular uptake of macromolecules Exp Biol Med (Maywood) 2008 233 94 105 10.3181/0704-RM-113 18156311 PMC2782745

[B19] Narang AS Thoma L Miller DD Mahato RI Cationic lipids with increased DNA binding affinity for nonviral gene transfer in dividing and nondividing cells Bioconjug Chem 2005 16 156 168 10.1021/bc049818q 15656587

